# P-356. Reduction of high-touch surface aerobic bacteria and fungi colony counts with the implementation of ActivePure continuous disinfection technology

**DOI:** 10.1093/ofid/ofae631.557

**Published:** 2025-01-29

**Authors:** Denise Taylor, Brittney Lynn, Kimberly Trosch, Daniel Marsh, Laurie Grier, Alexandre Malek

**Affiliations:** Ochsner LSU Health Shreveport, LA, USA, Shreveport, Louisiana; Ochsner LSU Health Shreveport, LA, USA, Shreveport, Louisiana; ActivePure Technologies, Dallas, TX, USA, Dallas, Texas; ActivePure Technologies, Dallas, TX, USA, Dallas, Texas; LSU Health Shreveport/ Ochsner LSU Health, Shreveport, Louisiana; Louisiana State University Health Sciences Center Shreveport, Shreveport, Louisiana

## Abstract

**Background:**

As hospitals nationwide rebound from the effects of the COVID pandemic, reduced staffing and increased hospital-acquired infections (HAIs) are still challenges. Surface disinfection plays a pivotal role in the prevention of HAIs. To improve high-touch surface disinfection and remove the dependence on the human factor, ActivePure continuous disinfection technology devices were installed in the Medical Intensive Care Unit (MICU) HVAC system at Ochsner LSU Health Shreveport in October 2023. ActivePure is a novel technique that kills microbes in the environment by using an advanced photocatalytic oxidation particle.

**Figure:**
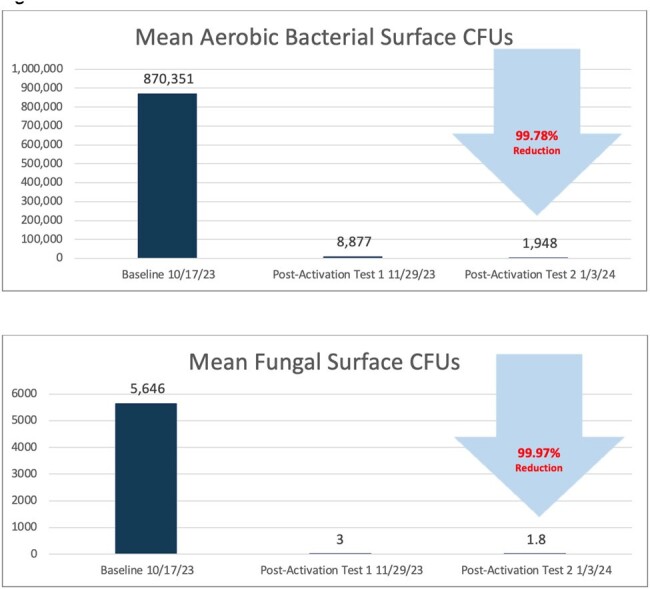

**Methods:**

A prospective study was conducted in the 19-bed MICU. Two weeks before device activation, 50 high-touch surface samples and 10 reservoir samples were collected by sterile sponge in situ and plated onto Nutritive (tryptic soy) agar for total aerobic microbial counts, Selective (Sabouraud dextrose) agar for total fungal counts, selective (MeReSa) agar for total MRSA counts, and CHROMagar selective for Carbapenem-Resistant *Acinetobacter baumannii* (CRAB). All agar plates were incubated at 30°C or 37°C (as applicable) for 48-96 hours. For evaluation, serial dilution plate counts were used to calculate the CFU per sponge for each media type. The detection limit was 10 CFU/sponge for each evaluation. After 4 weeks post-activation, 50 high-touch surface samples and 10 reservoir samples from the same pre-activation sample locations were collected and evaluated via the same methods, and the same was repeated at 9 weeks post-activation. Routine cleaning and disinfection protocols were unchanged during the entire study period.

Figure 2

**Figure:**
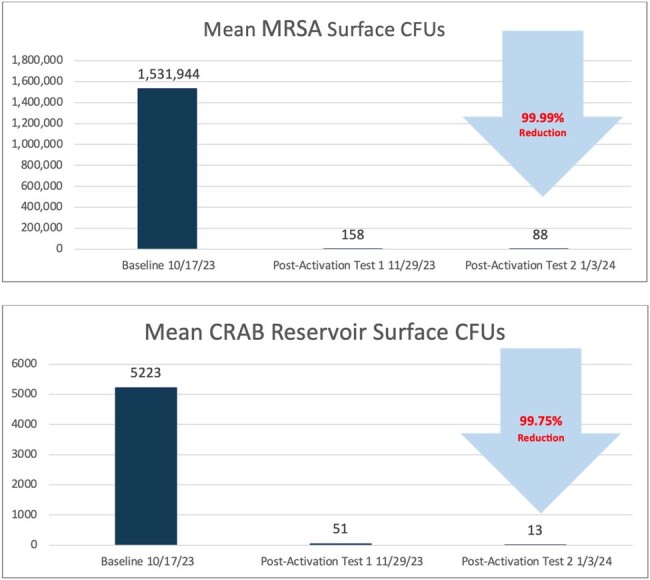

**Results:**

The mean aerobic bacterial and fungi surface CFUs were reduced over 99.78% (Fig 1). The mean MRSA surface CFUs were reduced by 99.99% and the mean CRAB surface CFUs were reduced by 99.75% (Fig 2). There was a 94% reduction in surfaces with greater than 500 CFUs/100cm^2^ of MRSA. There was a 100% reduction in reservoirs with greater than 500 CFUs/100cm^2^ of CRAB and 80% reduction in reservoirs with greater than 10 CFUs/100cm^2^ of CRAB.

**Conclusion:**

The ActivePure continuous disinfection technology devices significantly reduced total aerobic bacteria, including MRSA, difficult-to-treat CRAB, and fungi surface colony counts on high-touch surfaces.

**Disclosures:**

**Kimberly Trosch, RN, BSN**, ActivePure Technologies: Full time employee **Daniel Marsh, n/a**, ActivePure Medical: Advisor/Consultant

